# DHHC21 is a STIM1 protein S-acyltransferase that modulates immune function *in vivo*

**DOI:** 10.1242/jcs.264681

**Published:** 2026-09-11

**Authors:** Goutham Kodakandla, Ying Fan, Michael X. Zhu, Savannah J. West, Askar Akimzhanov, Darren Boehning

**Affiliations:** ^1^ Department of Biomedical Sciences, Cooper Medical School of Rowan University, Camden, NJ 08103, USA; ^2^ Department of Integrative Biology and Pharmacology, McGovern Medical School, University of Texas Health Sciences Center at Houston, Houston, TX 77030, USA; ^3^ Department of Biochemistry and Molecular Biology, McGovern Medical School, University of Texas Health Sciences Center at Houston, Houston, TX 77030, USA

**Keywords:** Store-operated Ca^2+^ entry, STIM1, Fas, Death receptor, Autoimmune lymphoproliferative syndrome, DHHC21, ZDHHC21, Protein S-acyltransferase

## Abstract

Depletion of Ca^2+^ from ER stores leads to the activation of Ca^2+^ channels on the plasma membrane known as store-operated Ca^2+^ entry. The proteins STIM1 and STIM2 function as ER Ca^2+^ sensors, and upon store depletion, they undergo a conformational change that allows them to bind to and gate Orai Ca^2+^ channels on the plasma membrane. We have shown that both Orai1 and STIM1 are dynamically S-acylated after store depletion, which is required for SOCE. These results suggest the requirement of a Ca^2+^-activated protein S-acyltransferase (PAT) such as DHHC21 (also known as zDHHC21). Here, we show that DHHC21 is essential for SOCE *in vitro* and *in vivo*. Using the *depilated* mouse model, which expresses DHHC21 that cannot be activated by Ca^2+^, we show that DHHC21 activation is a major mediator of STIM1 S-acylation and subsequent Ca^2+^ entry. Plasma membrane-localized DHHC21 is dynamically recruited into Orai1-STIM1 puncta upon store depletion, where it physically binds to STIM1. Finally, we show that *depilated* mice phenocopy many aspects of autoimmune lymphoproliferative syndrome (ALPS), including defective Fas-mediated Ca^2+^ release, T cell death, neutropenia and increased serum vitamin B12 levels. Targeting DHHC21 might be therapeutically beneficial for ALPS and diseases associated with deregulated activation of STIM1, such as tubular aggregate myopathy and Stormorken syndrome.

## Teaser

**Summary:** The protein acyltransferase DHHC21 S-acylates STIM1 *in vitro* and *in vivo* to regulate Ca^2+^ homeostasis and immune cell function.

## INTRODUCTION

Intracellular Ca^2+^ levels regulate many physiological functions. Store-operated Ca^2+^ entry (SOCE) is a mechanism where the depletion of endoplasmic reticulum (ER) Ca^2+^ stores leads to the activation of Ca^2+^ influx from the extracellular milieu (Berridge et al., 2000; Berridge, 1993; Berridge and Irvine, 1984; Putney, 1986; Lewis and Cahalan, 1989; Hoth and Penner, 1992; Parekh and Putney, 2005). The ER Ca^2+^ sensor stromal interaction molecule 1 (STIM1) is activated upon depletion of Ca^2+^ stores in the ER lumen and undergoes a conformational change leading to it approaching the plasma membrane (PM), where it binds to and activates a Ca^2+^ channel, Orai1 (Stathopulos et al., 2006; Kim and Muallem, 2011; Muik et al., 2011; Zhou et al., 2013). This intermolecular complex between STIM1 and Orai1 is termed the Ca^2+^ release-activated Ca^2+^ (CRAC) channel (Hoth and Penner, 1992; Hoth, 1995; Feske et al., 2005). CRAC channels form macromolecular complexes near ER–PM junctions, which constitute the nexus of SOCE. These complexes have been described as ‘puncta’ owing to the punctate appearance in microscopic images (Luik et al., 2006). Abnormalities in CRAC channel function lead to disorders in the immune and musculoskeletal systems, such as severe combined immunodeficiency (SCID), tubular aggregate myopathy and Stormorken syndrome (Feske et al., 1996, 2005, 2006, 2010; Feske, 2007; McCarl et al., 2009).

The mechanisms by which STIM1 and Orai1 proteins colocalize near ER–PM subdomains upon store-depletion have been a long-standing debate in the field. One prominent hypothesis is the diffusion trap model, which postulates a stochastic binding of activated STIM1 to Orai1 in these subdomains, where STIM1 ‘traps’ Orai1 to induce SOCE (Hoover and Lewis, 2011; Wu et al., 2014). However, this model does not explain the active targeting of STIM1 to phosphatidylinositol 4,5-bisphosphate (PIP2) domains in membrane lipid rafts (Calloway et al., 2011). In addition, studies have also shown that both Orai1 and STIM1 exist in dynamic equilibrium, where these proteins can transfer between different puncta (Wu et al., 2014). We have shown that S-acylation of Orai1 and STIM1 regulates CRAC channel formation (West et al., 2022b; Kodakandla et al., 2022, 2023). S-acylation is the reversible addition of lipid moieties to intracellular cysteine residues of target proteins (Chamberlain and Shipston, 2015). S-acylation is mediated by a set of enzymes known as palmitoyl acyltransferases (PATs). PATs are also known as DHHC (also known as zDHHC) enzymes owing to the presence of an aspartate-histidine-histidine-cysteine motif in the active site of these enzymes (Mitchell et al., 2006; Jennings and Linder, 2012). S-acylation affects protein stability, trafficking to subcellular compartments and shuttling between membrane subdomains (Kabouridis et al., 1997; Melkonian et al., 1999; Resh, 1999; Rocks et al., 2005; Levental et al., 2010). We have shown that S-acylation actively targets Orai1 and STIM1 to puncta, providing an additional level of regulation to SOCE (Kodakandla et al., 2022; West et al., 2022b). Our findings warrant refinement of the diffusion trap model.

Several DHHC enzymes are active in the PM, including DHHC5, DHHC20 and DHHC21 (also known as zDHHC5, zDHHC20 and zDHHC21, respectively) (Ohno et al., 2006; Chen et al., 2020; Fan et al., 2020; Carreras-Sureda et al., 2021). Previously, we have shown that DHHC5 plays a crucial role in cardiomyocyte β-adrenergic signaling by targeting Gα proteins (Chen et al., 2020). More importantly, we have shown that DHHC21 is essential for T cell Ca^2+^ signaling by S-acylating components of both the Fas and the T cell receptor (TCR) complexes (Fan et al., 2020; Akimzhanov et al., 2010). We also exploited the mouse model *depilated*, which has an in-frame deletion of a single phenylalanine residue in DHHC21 that eliminates a calmodulin-binding site (Fan et al., 2020; Bieerkehazhi et al., 2022). *Depilated* mice have significant deficits in T cell signaling and differentiation into effector T cell lineages. *In vitro*, T cells from *depilated* mice have defective TCR signaling, including impaired activation of Lck, PLC-γ1, ZAP-70 and ERK pathways after TCR ligation (Bieerkehazhi et al., 2022; West et al., 2022a). Ca^2+^ transients induced by TCR ligation are significantly reduced in T cells from *depilated* mice, likely due to decreased S-acylation and activation of proteins such as Lck (Fan et al., 2020). The full repertoire of T cell signaling proteins regulated by DHHC21 S-acylation remains to be elucidated.

Based upon our previous findings in *depilated* mice, in this study, we examined the hypothesis that DHHC21 S-acylates STIM1. We show that STIM1 S-acylation is significantly abrogated in spleens from homozygous *depilated* mice. This is associated with defects in splenocyte SOCE. We also show that DHHC21 is recruited into STIM1 puncta, and this is altered in *depilated* splenocytes. Consistent with the requirement of DHHC21 for Fas-mediated Ca^2+^ release and cell death of T cells, we show that *depilated* mice phenocopy autoimmune lymphoproliferative syndrome (ALPS). We conclude that DHHC21 is a major PAT for STIM1 *in vitro* and *in vivo*, and that *depilated* mice are a possible model for ALPS.

## RESULTS

### DHHC21 is required for STIM1 S-acylation

**Fig. 1.**
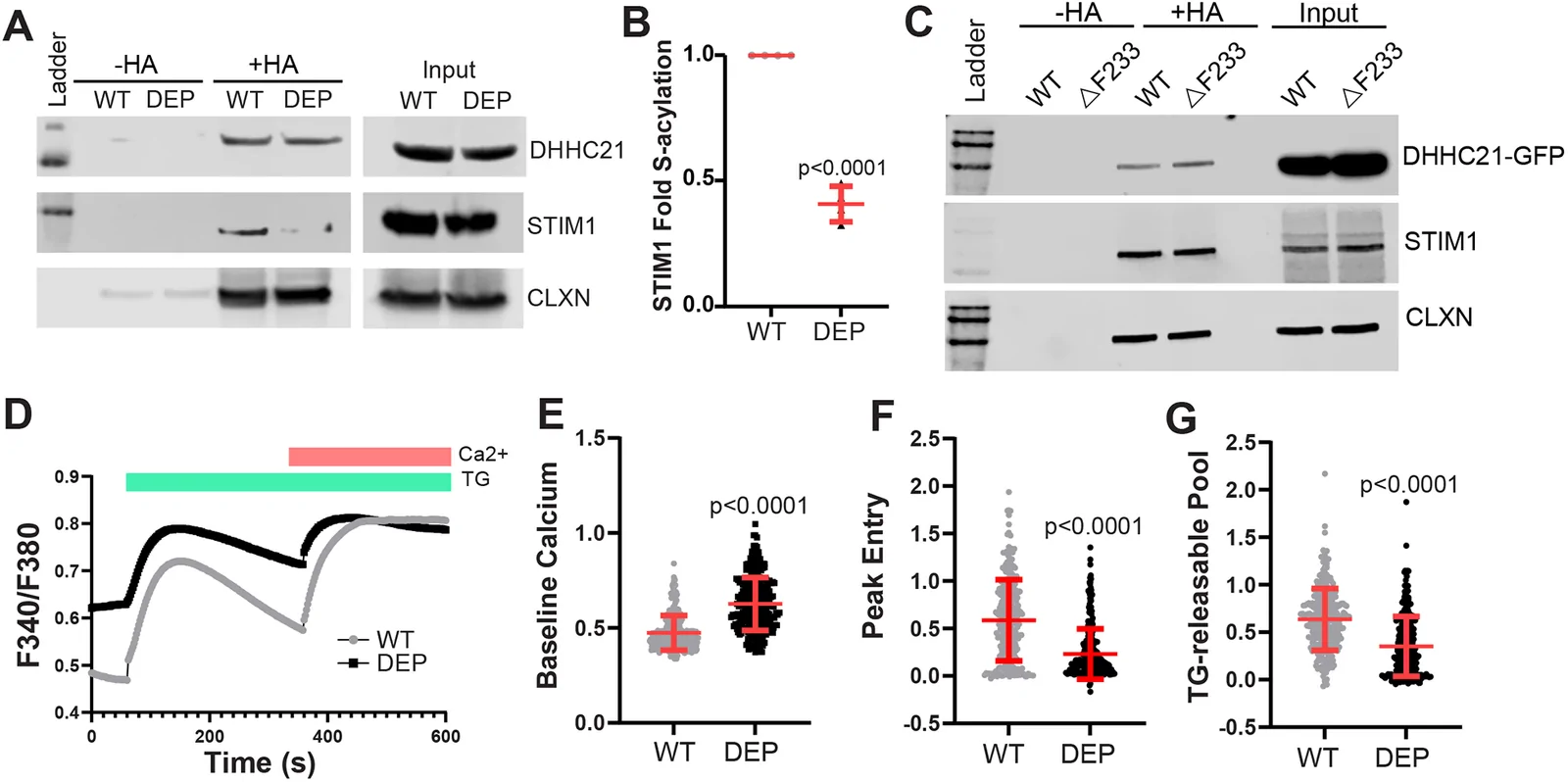
**DHHC21 is the STIM1 protein acyltransferase and regulates store-operated Ca^2+^ entry.** (A) Spleens from C57BL6F1/J WT and *depilated* (DEP) homozygous mice were collected, and acyl-RAC was performed. Reactions without hydroxylamine (−HA) are a negative control. Calnexin was used as a loading control for S-acylated proteins. (B) Quantification of fold S-acylation of STIM1 normalized to calnexin from four independent experiments. Statistical significance between the groups was determined using a two-tailed unpaired Student's *t*-test. Error bars indicate s.d. (C) WT and ΔF233 DHHC21–GFP plasmids were co-expressed along with WT STIM1–mRFP and WT Orai1–Myc in DHHC21-KO cells, followed by acyl-RAC. (D–G) Splenocytes were collected from C57BL6 WT and *depilated* homozygous mice and used for Fura2 imaging using 10 μM thapsigargin (TG) for store-depletion and 1 mM Ca^2+^ addback to assess store-operated Ca^2+^ entry. F340/F380 ratios were used to quantify Ca^2+^ levels in these cells. Representative traces are shown in D. Baseline Ca^2+^ levels (E), peak entry (F), and TG-releasable pool (G) were calculated from the F340/F380 values. Total number of cells analyzed: 280 WT, 298 DEP. Statistical significance between the groups was determined using a two-tailed unpaired Student's *t*-test. Error bars indicate s.d.

We have previously shown that CRAC channel components Orai1 and STIM1 are S-acylated (Kodakandla et al., 2022; West et al., 2022b). *Depilated* mice have an in-frame deletion of phenylalanine 233 (F233) residue in the C-terminal tail of DHHC21 that eliminates calmodulin binding (Bieerkehazhi et al., 2022; Fan et al., 2020). This mutation leads to deficits in TCR signaling and T cell differentiation. We concluded that the ΔF233 mutation is likely a loss-of-function mutation (Bieerkehazhi et al., 2022; Fan et al., 2020). To determine whether *depilated* mutant mice have deficits in STIM1 S-acylation *in vivo*, we harvested spleens from wild-type and *depilated* mice and performed an acyl-RAC assay to purify S-acylated proteins. We found that STIM1 has significantly reduced S-acylation in *depilated* spleen (Fig. 1A,B). The S-acylation of DHHC21 and the ER protein calnexin was unchanged. Total levels of all three proteins were similar in wild-type and homozygous mutant mice. Overexpression of wild-type or ΔF233 DHHC21 in HEK cells expressing endogenous DHHC21 did not significantly affect S-acylation of STIM1, indicating that the ΔF233 mutant enzyme does not function as a dominant-negative inhibitor of the wild-type protein (Fig. 1C). We next determined whether SOCE was altered in wild-type (WT) and homozygous *depilated* splenocytes. Resting (baseline) Ca^2+^ levels were significantly higher in *depilated* homozygous splenocytes, indicating significant alterations in Ca^2+^ homeostasis (Fig. 1D,E). To measure SOCE, we depleted ER stores with thapsigargin (TG) in a Ca^2+^-free solution and then added back Ca^2+^ to measure entry (Fig. 1D). We found that homozygous *depilated* splenocytes had significantly reduced SOCE (Fig. 1F) and a smaller TG-releasable pool (Fig. 1G). The smaller ER Ca^2+^ store size revealed by TG indicates that the higher cytosolic resting Ca^2+^ in depilated splenocytes might be due to either altered sarcoendoplasmic reticulum Ca^2+^ ATPase (SERCA) activity or increased IP_3_R activity. These results indicate that DHHC21 is a major PAT for STIM1 *in vitro* and *in vivo*, and is necessary for efficient SOCE.

### DHHC21 is a major contributor to store-operated Ca^2+^ entry

**Fig. 2.**
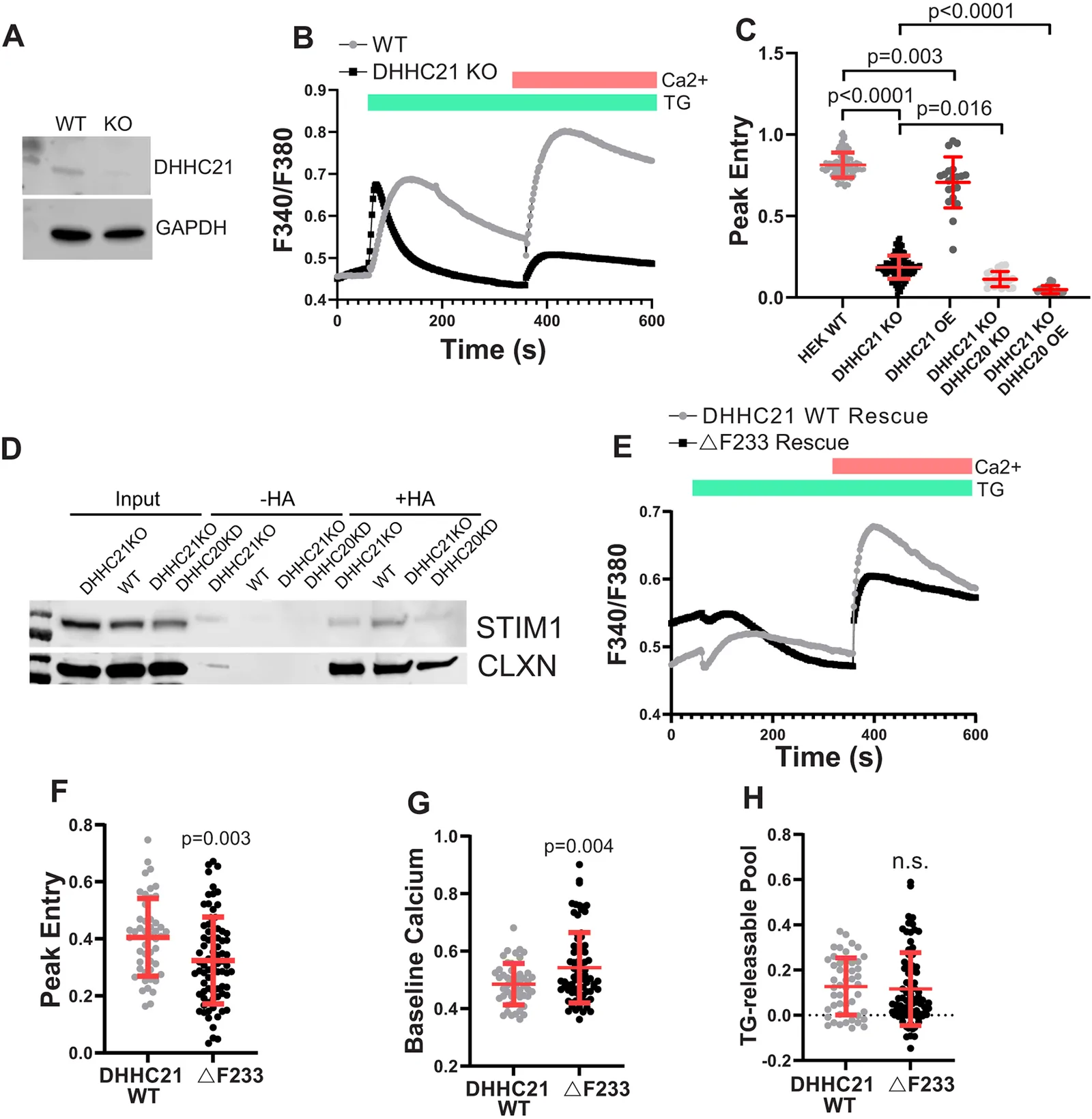
**DHHC21 KO cells have impaired SOCE.** Clonal DHHC21 CRISPR KO HEK293 cells were generated as described in the Materials and Methods section. (A) Western blotting was performed on whole-cell lysates obtained from WT and DHHC21-KO cells and probed for DHHC21. GAPDH was used as a loading control. Blot shown representative of three independent repeats. (B) Fura2 imaging was performed on WT and DHHC21-KO cells. Store depletion was induced by 10 μM thapsigargin (TG) in Ca^2+^-free buffer. Ca^2+^ (1 mM) was added back after 6 min to allow Ca^2+^ entry. (C) Peak Ca^2+^ entry in WT, DHHC21-KO, DHHC21 overexpression (DHHC21-OE), DHHC21-KO+DHHC21 knockdown (DHHC20-KD), and DHHC21-KO+DHHC20-OE cells. Statistical significance between the groups was determined using one-way ANOVA, followed by Tukey's post hoc analysis. Error bars indicate s.d. The total number of cells analyzed was: 108 WT, 117 DHHC21-KO, 117 DHHC21-OE, 20 DHHC20-KD+DHHC21-KO, and 17 DHHC20-OE+DHHC21-KO. (D) DHHC20 was knocked down in DHHC21-KO cells, and acyl-RAC was performed as detailed in the Materials and Methods section. A reaction without hydroxylamine (−HA) was used as a negative control. Calnexin was used as a loading and positive control. Blot shown representative of three independent repeats. Input, 5% of total lysate. (E–H) Wild-type or ΔF233 DHHC21–eGFP was co-expressed with STIM1–mRFP and Orai1–Myc in DHHC21-KO cells, and Fura2 imaging was performed. Store-operated Ca^2+^ entry was determined as in B. Representative traces are shown in E. Peak Ca^2+^ entry (F), baseline Ca^2+^ (G), and the TG-releasable Ca^2+^ pool (H) were quantified for WT and ΔF233 cells. The total number of cells analyzed was as follows (48 WT, 77 F233). Statistical significance between the groups was determined using two-tailed unpaired Student's *t*-test. Error bars indicate s.d.

To determine the role of DHHC21 in SOCE, we generated a DHHC21 CRISPR knockout (KO) HEK293 cell line. We confirmed a complete loss of expression by western blotting (Fig. 2A). We found that DHHC21-KO cells had significantly reduced SOCE induced by TG (Fig. 2B,C). Conversely, overexpression of DHHC21 in WT cells did not increase SOCE (Fig. 2C). It has been shown that DHHC20 can S-acylate Orai1 (Carreras-Sureda et al., 2021). We found a small but statistically significant further reduction of SOCE when knocking down DHHC20 expression with siRNA, indicating partial redundancy between the two enzymes. However, we were not able to rescue SOCE by overexpressing DHHC20 in DHHC21 KO cells (Fig. 2C). To complement the Ca^2+^ imaging data, we performed Acyl-RAC to determine if STIM1 acylation was affected by DHHC21 KO. We found that STIM1 S-acylation was reduced in KO cells and, consistent with the Ca^2+^ imaging data, was further reduced when DHHC20 was knocked down in the KO cells (Fig. 2D). Thus, both *in vitro* and *in vivo*, DHHC21 is a STIM1 S-acylating enzyme. We next rescued DHHC21 KO cells by expressing WT and ΔF233 DHHC21-eGFP. Expression of WT DHHC21 partially rescued SOCE in DHHC21 KO cells. The ΔF233 DHHC21 enzyme only partially restored SOCE, showing significantly less effective rescue than WT (Fig. 2E,F). Interestingly, this *in vitro* rescue model with ΔF233 in a null DHHC21 background recapitulated the increased baseline Ca^2+^ levels found in *depilated* splenocytes (Fig. 2G). This suggests that ΔF233 has direct effects on baseline Ca^2+^ independent of a complete loss of function. Finally, we found that the TG-releasable pool was similar between WT and ΔF233 rescue (Fig. 2H). This finding is inconsistent with what we found in splenocytes and might be related to the different Ca^2+^-handling machinery in HEK cells. We conclude in this independent model that DHHC21 is a central mediator of SOCE, and the *depilated* ΔF233 mutation cannot fully rescue DHHC21 function. The DHHC20 enzyme also regulates STIM1 S-acylation but cannot rescue SOCE in DHHC21 KO cells. This might represent a distinct role in STIM1 regulation, or alternatively, that overexpressed DHHC20 does not recapitulate the function of the endogenous enzyme. The other major plasma membrane DHHC enzyme, DHHC5, has a unique substrate profile and is expressed primarily in the heart and nervous system (Chen et al., 2020; Li et al., 2010). It has not been shown to be a major mediator of store-operated Ca^2+^ entry. As such, it was not tested in this study.

### Co-localization of WT and ΔF233 DHHC21 with STIM1

**Fig. 3.**
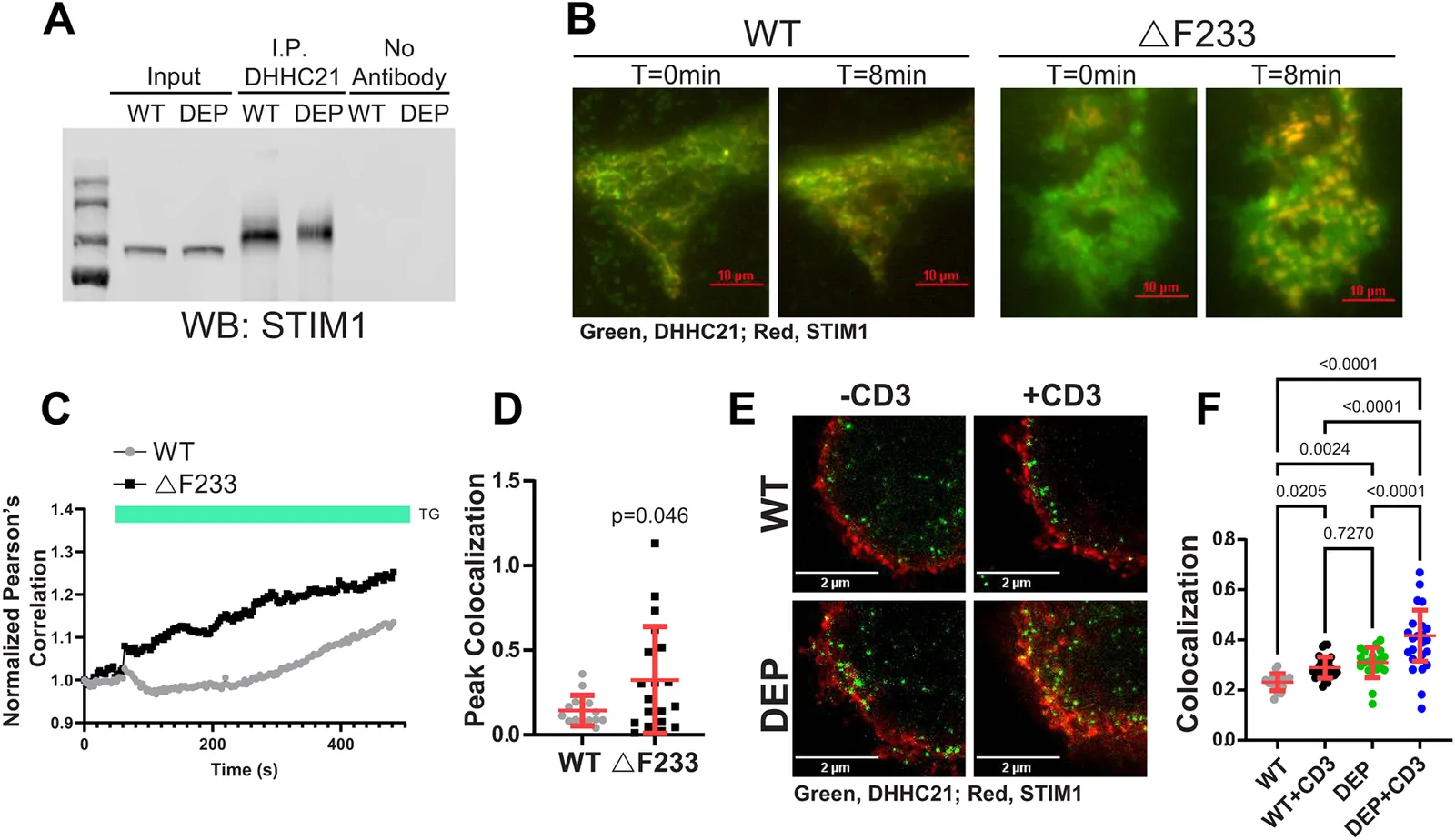
**Store depletion increased colocalization of DHHC21 with STIM1.** (A) Co-immunoprecipitation (I.P.) of STIM1 by DHHC21 from WT and *depilated* (DEP) splenocytes. Blot shown representative of three independent repeats. Input, 5% of total lysate. (B) DHHC21-KO HEK293 cells were transfected with WT or ΔF233 DHHC21–eGFP, STIM1–mRFP and Orai1–Myc plasmids and store-depletion was induced using 10 μM thapsigargin (TG). Representative merged images are shown for a single experiment at times zero and at eight minutes after TG treatment. (C,D) Peak colocalization was quantified using Pearson's correlation values obtained from WT and ΔF233-expressing cells. Statistical significance between the groups was determined using a two-tailed unpaired Student's *t*-test. Error bars in D indicate s.d. The total number of cells analyzed was: 14 WT, 19 F233. (E) Splenocytes were obtained from C57BL6F1/J WT and *depilated* homozygous mice and used for STED imaging. Store depletion was induced by anti-CD3 antibodies. (F) Pearson's correlation between DHHC21 and STIM1 before and after store depletion. The total number of cells analyzed was: 18 WT, 24 WT+CD3, 20 DEP, 23 DEP+CD3. Significance was determined using a one-way ANOVA followed by Tukey's post-hoc analysis for multiple comparisons. Error bars indicate s.d.

If DHHC21 is a protein S-acyltransferase for STIM1, the two proteins should interact (at least transiently). We made lysates from the spleens of WT and homozygous *depilated* mice, and co-immunoprecipitated STIM1 with DHHC21. Interestingly, STIM1 binds avidly to DHHC21 in spleens from both WT and *depilated* mice in co-immunoprecipitation experiments (Fig. 3A). We hypothesized that binding of DHHC21 to STIM1 might increase after store depletion. To test this hypothesis, we performed total internal reflection fluorescence (TIRF) imaging of DHHC21–GFP and STIM1–mRFP in DHHC21 KO cells. As shown in Figs 3B–D, Fig. S1, and Movies 1 and 2, store depletion leads to rapid recruitment of both WT and ΔF233 DHHC21 into STIM1 puncta. We observed a significantly higher peak colocalization of ΔF233 DHHC21 compared to WT DHHC21. We confirmed that the endogenous DHHC21 and STIM1 proteins in HEK293 cells bind after store depletion using superresolution imaging (Fig. S2). To determine the recruitment of DHHC21 to STIM1 in splenocytes, we performed superresolution imaging on splenocytes obtained from WT and *depilated* mice. We treated splenocytes *in vitro* with anti-CD3 antibody to activate TCR signaling and SOCE prior to fixation and staining for DHHC21 and STIM1. As shown in Fig. 3E,F, we detected a significantly higher colocalization between DHHC21 and STIM1 in splenocytes treated with anti-CD3. The colocalization of DHHC21 and STIM1 in *depilated* mice was even more pronounced, corroborating our findings by TIRF imaging in the *in vitro* model. Together, these results indicate that ΔF233 DHHC21 binds STIM1 more avidly than the WT protein after store depletion. We interpret these findings to mean that ΔF233 DHHC21 binds STIM1 but does not release the protein because it cannot complete the enzymatic cycle. These results also suggest that Ca^2+^-calmodulin binding to DHHC21 regulates enzymatic activity independently of substrate binding.

### *Depilated* mice phenocopy ALPS

**Fig. 4.**
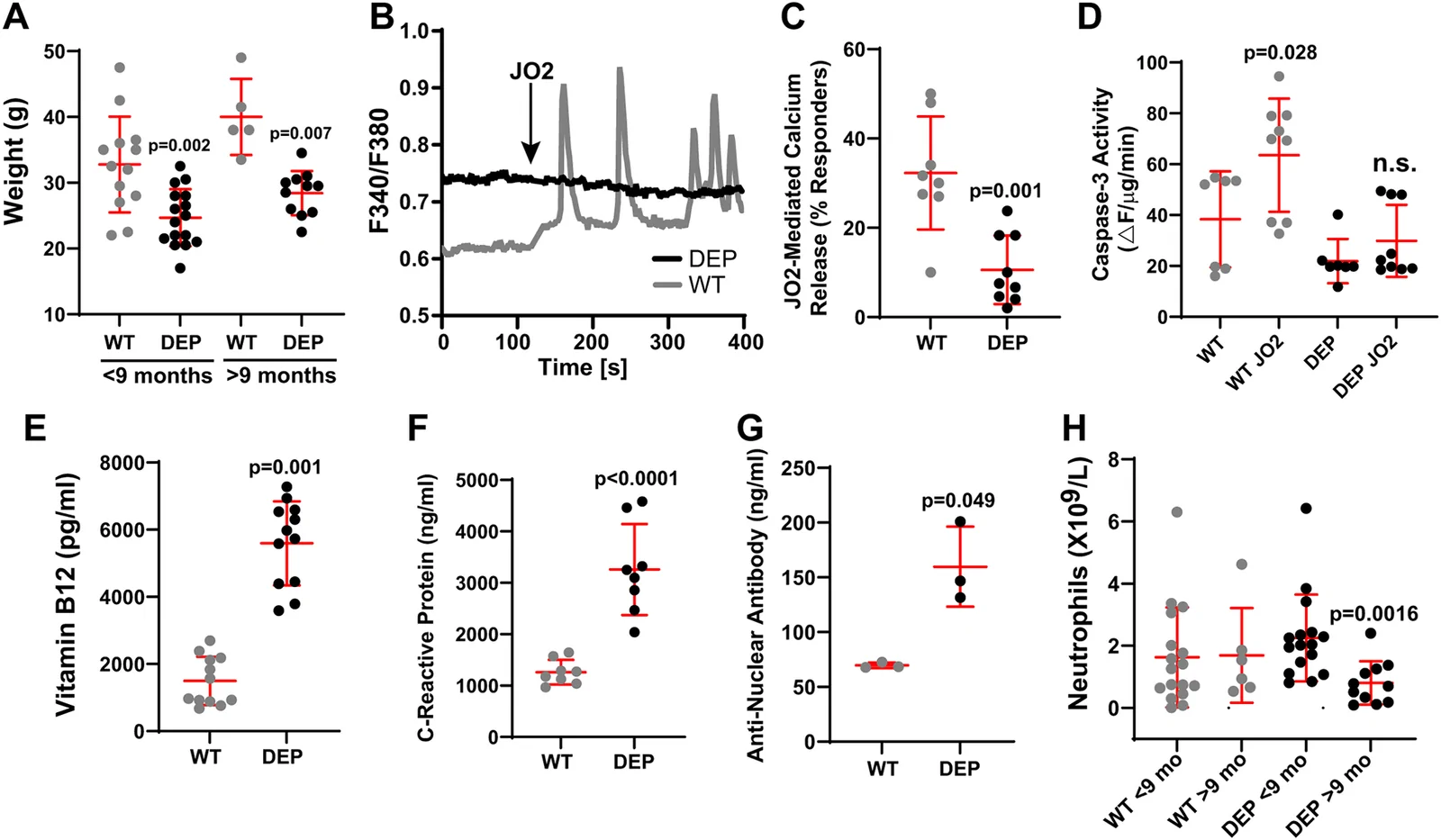
***Depilated* mice phenocopy ALPS.** (A) WT and *depilated* (DEP) mice younger and older than 9 months were weighed and analyzed using a two-tailed unpaired Student's *t*-test. Error bars represent s.d. The total number of mice analyzed was: 18 WT, 26 DEP. (B,C) Splenocytes from C57BL6F1/J WT and *depilated* homozygous mice were isolated, and Fura2 Ca^2+^ imaging was performed. Representative splenocyte responses to stimulation with the Fas agonistic antibody JO2 (B) and quantification of the percentage responders to JO2 averaged from eight (WT) and nine (*depilated*) mice (C) are shown. (D) Caspase-3 enzymatic activity with and without JO2 in WT and *depilated* splenocytes. (E–G) Serum vitamin B12 (E), C-reactive protein (F) and anti-nuclear antibody (G) levels in WT and *depilated* mice. (H) Neutrophil counts in WT and *depilated* mice younger and older than 9 months. Data were analyzed using a two-tailed unpaired Student's *t*-test (age was used as the independent variable for this analysis). Error bars represent s.d.

We have previously shown that Fas signaling in T lymphocytes requires Ca^2+^ release (Akimzhanov and Boehning, 2015; Akimzhanov et al., 2010). Engagement of the Ca^2+^ release machinery requires rapid and dynamic S-acylation of signaling proteins such as the tyrosine kinase Lck (Akimzhanov and Boehning, 2015). Pathogenic variants in the Fas pathway can cause ALPS (Casamayor-Polo et al., 2021). The primary diagnostic criteria for ALPS are chronic lymphadenopathy and splenomegaly, increased peripheral CD4^−^/CD8^−^ T cells, defective Fas-mediated lymphocyte apoptosis and a pathogenic variant in a Fas pathway gene (Matson and Yang, 2020). Secondary criteria include increased serum vitamin B12 levels, autoimmune cytopenia, and increased IgG levels. However, ∼10–20% of individuals with ALPS have no known genetic mutation (ALPS-U), suggesting that additional unknown genes contribute to disease progression (Palmisani et al., 2023). We have previously shown that *depilated* mice have marked lymphadenopathy and splenomegaly (Bieerkehazhi et al., 2022). In addition, we discovered profound defects in T cell development in these mice (Bieerkehazhi et al., 2022). We have also shown, using shRNA-mediated knockdown, that DHHC21 is required for Fas-mediated Ca^2+^ release (Akimzhanov and Boehning, 2015). Thus, we hypothesize that *depilated* mice might phenocopy ALPS. *Depilated* mice demonstrate a failure to thrive as determined by weight in both young and adult mice (Fig. 4A). We found that splenocytes isolated from homozygous *depilated* mice had significant defects in both Fas-mediated Ca^2+^ release and apoptotic cell death as determined by caspase-3 enzymatic activity (Fig. 4B–D). This was associated with significantly elevated serum vitamin B12 levels (Fig. 4E). C-reactive protein (CRP), a marker of inflammation and autoimmunity, was also elevated in the sera of homozygous mutant mice (Fig. 4F). Similarly, we found a significant elevation of the autoimmunity marker anti-nuclear antibody (ANA) in the sera of homozygous mice (Fig. 4G). Finally, we found age-dependent neutropenia in homozygous mutant mice (Fig. 4H). We conclude that *depilated* mice phenocopy ALPS and suggest that mutations in *DHHC21* should be investigated in ALPS-U individuals without a mutation in a known causative gene.

## DISCUSSION

S-acylation plays a crucial role in store-operated Ca^2+^ entry by modifying the localization and activity of Orai1 and STIM1. Previously, we have shown that both Orai1 and STIM1 undergo S-acylation upon store depletion. S-acylation directs these proteins to membrane subdomains where they form CRAC channels to promote SOCE. Cysteine mutant versions of these proteins that cannot undergo S-acylation show deficits in SOCE. Previously, another group demonstrated that DHHC20 S-acylates Orai1 (Carreras-Sureda et al., 2021). In this work, we show that DHHC21 S-acylates STIM1 *in vivo* using *depilated* mice that have the ΔF233 mutation in DHHC21. We also show that ΔF233 DHHC21 can still bind but cannot S-acylate STIM1. This leads to significant deficits in SOCE and immune system function *in vivo*. It remains to be determined whether Orai1 can also be S-acylated by DHHC21 or whether two different PATs are required for SOCE after store depletion.

DHHC21 is a plasma membrane-localized protein S-acyltransferase. We have shown previously that DHHC21 plays a key role in S-acylation of many proteins involved in TCR signaling. TCR components such as PLC-γ1, Lck, and ZAP-70 undergo S-acylation upon treatment with anti-CD3, which activates the TCR (Fan et al., 2020). We have previously shown that Jurkat cells with DHHC21 knocked down by shRNA or CD4+ T cells isolated from *depilated* mice are defective in S-acylation of these proteins and downstream signaling events, indicating that DHHC21 plays a prominent role in TCR signaling (Bieerkehazhi et al., 2022; Fan et al., 2020; Akimzhanov and Boehning, 2015). This formed the basis for our hypothesis that DHHC21 might mediate the S-acylation of STIM1. The S-acylation of STIM1 by PM-localized DHHC21 strongly suggests that S-acylation of the STIM1 tail stabilizes its PM association. The polybasic domain of STIM1 has been shown to interact with membrane phospholipids such as PIP2 and physically bind to the PM (Cohen et al., 2023). In addition, isoleucine 384 (I384) in the cholesterol-binding site within the SOAR-CAD domain of STIM1 has also been shown to bind to the PM upon store-depletion induced by TG. This interaction was shown to enhance SOCE (Pacheco et al., 2016). Together, these domains stabilize the active conformation of STIM1. The redundant C-terminal domains mediating PM binding of STIM1 might explain the partial SOCE retained in *depilated* splenocytes and DHHC21 KO HEK293 cells. Further experiments are warranted to determine the mechanism(s) by which SOCE is partially retained in *depilated* splenocytes.

We observed differences in SOCE in *depilated* splenocytes compared to CRISPR-KO HEK293 cells (Figs 1 and 2). In addition, rescue experiments demonstrated that ΔF233 DHHC21 expression has direct effects on baseline Ca^2+^ in KO cells, indicating that this mutant enzyme has significant consequences for Ca^2+^ homeostasis (Fig. 2F). We conclude that ΔF233 DHHC21 is not a complete loss of function. Indeed, we found that this enzyme can bind STIM1 even more avidly than the WT enzyme (Fig. 3). This was somewhat unexpected. We hypothesize that DHHC21-ΔF233 is still competent to bind substrate (including STIM1), but it can no longer release the substrate because of a lack of enzymatic activity. It is likely that ΔF233 DHHC21 sequesters additional substrates at the PM with significant ramifications for cellular physiology. Recently, a mouse model with a T-cell-specific KO of DHHC21 has been described (Meng et al., 2025). Like our previous studies on T cell development and differentiation in *depilated* mice (Fan et al., 2020; Bieerkehazhi et al., 2022), the researchers found significant effects on the peripheral T cell compartment, but with many important differences. Perhaps most strikingly, they found that both spleen and lymph node sizes were smaller with fewer total cell counts in contrast to the splenomegaly and lymphadenopathy found in *depilated* mice. Thus, their findings support our hypothesis that ΔF233 DHHC21 is not a loss-of-function mutation and, indeed, might have significant gain-of-function effects on T cell signaling. It was found that 21 proteins in T cells are likely substrates of DHHC21 using proximity labeling (Meng et al., 2025), all of which could potentially be sequestered by ΔF233 DHHC21 to affect T cell function. Regardless of the functional effect of the ΔF233 mutation, we can make a few strong conclusions. Eliminating the DHHC21 gene in cultured cells causes a large loss in SOCE and STIM1 S-acylation, and the ΔF233 mutation cannot fully rescue this phenotype. We also found that the DHHC21 enzyme binds to STIM1 and is actively recruited into puncta during store depletion. The recruitment of DHHC21 to CRAC puncta necessitates its regulation of SOCE. Finally, splenocytes from depilated mice have a phenotype similar (but not identical) to DHHC21-KO HEK cells. It is important to note that the DHHC21 ΔF233 mutation does not affect the active site but rather eliminates its regulation by calmodulin (Bieerkehazhi et al., 2022). We provided one possible interpretation (substrate sequestration), but it could also be that the enzyme is still functional (and S-acylates substrates) but no longer activated in a stimulus-dependent manner. Future work will confirm this hypothesis and further explore the ALPS-like phenotype in depilated mice.

## MATERIALS AND METHODS

### Cells, antibodies and constructs

Authenticated HEK293T cells were purchased from American Type Culture Collection (ATCC; cat. no. CRL-3216) and cultured in Dulbecco's modified Eagle's medium (DMEM; Corning, 10-013-CV) supplemented with 10% fetal bovine serum (FBS), 1% L-glutamine (Corning, 25-005-CI), and 1% penicillin-streptomycin (Gibco 15140122). DHHC21-KO cells were generated using CRISPR-Cas9 plasmids obtained from Genscript Inc. The sgRNA sequences used in these plasmids for KO of DHHC21 are 5′-AAGTGGTAGGGAACTCGCAG-3′ and 5′-ATGAGACTAGCAGCCTTTAT-3′. KO clones were isolated by GFP selection followed by serial dilution to isolate isogenic clones. KO of DHHC21 was confirmed by western blotting. For experimental manipulations, cells were plated on polystyrene tissue culture dishes or six-well plates. For imaging, cells were plated on poly-L-lysine-coated coverslips in six-well plates. Cells were transfected with 0.5 μg STIM1 and 0.5 μg DHHC21 plasmids (see below) per 35-mm dish. All cells were maintained at 37°C and 5% CO_2_ until use. All cell lines were periodically checked for contamination using the ATCC mycoplasma testing service. Antibodies for immunoblotting and immunofluorescence imaging were purchased from commercially available sources: STIM1 (cat. no. 4961S), calnexin (cat. no. 2679), anti-rabbit immunoglobulin, horseradish peroxidase-linked secondary antibody (cat. no. 7074S), anti-mouse immunoglobulin, and horseradish peroxidase-linked secondary antibody (cat. no. 7076P2) were from Cell Signaling Technology; anti-zDHHC21 (PA525096) was from Thermo Fisher Scientific; anti-CD3 human antibody (cat. no. 14-0037-82) was from eBiosciences. JO2 antibody (cat. no. 554255) was from BD Biosciences. The secondary antibodies for super-resolution STED imaging were obtained from Abberior Inc (STRED-1001, STRGREEN-1001). DHHC21–eGFP plasmids were purchased from Genscript Inc. The DHHC21–GFP plasmid was constructed by VectorBuilder. The DHHC21–FLAG plasmid was a kind gift from Dr Masaki Fukata (National Institute for Physiological Sciences, Japan). The DHHC21-ΔF233 mutant was generated using q5 site-directed mutagenesis kit from New England Biolabs using primers (forward 5′-TCAGAAGTTTTTGGCACTCGTTG-3′ and reverse 5′-GGTCTGCTGCCATGGCTT-3′). Mutagenesis was confirmed using Sanger Sequencing (Eton Biosciences). STIM1–mRFP was a generous gift from David Holowka and Barbara Baird (Cornell University, New York, USA). Thiol-Sepharose beads used for acyl-RAC were obtained from Nanocs Inc. and activated according to the manufacturer's protocol before continuing with the acyl-RAC protocol listed below. All other chemicals and reagents were purchased from Sigma-Aldrich or VWR unless otherwise indicated.

### Mice

WT and *depilated* (Zdhhc21^dep^) mice in the C56BL/6 background were bred in our barrier vivarium under pathogen-free conditions in accordance with the recommendations in the Guide for the Care and Use of Laboratory Animals of the National Institutes of Health. The animals were handled according to the animal care protocol #2020-1252 approved by the Rowan University Institutional Animal Care and Use Committee. Homozygous *depilated* mice had a higher mortality rate before the age of 4 weeks. To decrease the mortality rate, DietGel Boost was provided after birth as a high-calorie dietary supplement to the pups until they were weaned at 4 weeks of age. A quantitative (q)PCR strategy was used to genotype pups. *Depilated* mice were also confirmed visually by the loss of hair. Neutrophil counts were determined using a Zoetis Diagnostics Vetscan HM5 in male and female mice ranging from 4–52 weeks old. Mice used for other experiments were males and females, ranging in age from 6 to 8 weeks.

### Acyl-RAC assay

The acyl-RAC assay was performed as previously described by our group (Tewari et al., 2020). Cell and tissue lysates were collected in lysis buffer [1% dodecyl-β-D-maltoside in DPBS, supplemented with cOmplete protease inhibitor cocktail (Roche), 10 μM ML211 (acyl protein thioesterase inhibitor) and 10 mM PMSF]. The lysates were centrifuged at 4°C for 30 min at 20,000 ***g***. 500 μg of protein lysate were used for enrichment of the S-acylated proteome. Protein precipitation was performed using 2:1 methanol:chloroform, and the protein pellets were incubated with 0.1% methylmethanethiosulfonate (MMTS) for 20 min at 42°C. Excess MMTS in the protein lysates was removed by three rounds of protein precipitation using methanol and chloroform. The resulting protein pellets were dissolved in 2SHB buffer (2% SDS, 5 mM EDTA, 100 mM HEPES, pH 7.4). 5% of the final volume was saved for input, and the remaining was used for acyl-RAC. 400 mM hydroxylamine (HA) was added to protein lysates to cleave the thioester bonds at pH 7. The lysates were incubated with thiol-Sepharose resin with rotation overnight at 4°C. The next day, the samples were washed using wash buffer (1% SDS, 5 mM EDTA, 100 mM HEPES, pH 7.4). Proteins were eluted using 10 mM DTT in SDS buffer (1% SDS, 50 mM Tris-HCl, 10% glycerol, and 1% Bromophenol Blue) at 80°C for 15 min with continuous shaking. Samples were then resolved on 10% SDS-PAGE gels and analyzed using western blotting.

### Western blotting

Proteins were separated on freshly made SDS polyacrylamide (SDS-PAGE) gels with 10% resolving and 4% stacking gels. After electrophoresis, proteins were transferred to nitrocellulose membranes. The membranes were blocked with 5% non-fat milk in 0.1% TBST (Tris-buffered saline with 0.1% Tween 20) for 1 h. After 1 h, the membranes were briefly rinsed in TBST and incubated with primary antibodies overnight at 4ºC. Next day, the membranes were washed four times at room temperature for 30 min with TBST, followed by incubation with HRP-conjugated secondary antibodies for 2 h at room temperature. Finally, the membranes were washed in TBST four times for 30 min and developed using SuperSignal electrochemiluminescence (ECL) (Thermo Fisher Scientific) and imaged using a LiCor imager. LiCor Image Studio was used to make brightness and contrast adjustments as needed. Primary antibody dilutions were as follows: STIM1, 1:2000; DHHC21, 1:1000; calnexin, 1:2000. Secondary antibodies were diluted 1:2500. All dilutions were done using TBST.

### Co-immunoprecipitation

Protein lysates were obtained from the spleens of WT and *depilated* mice using the lysis buffer used in the acyl-RAC section above. Protein lysates (1 mg) were incubated with 1.5 μg of anti-zDHHC21 polyclonal antibody (PA525096; Thermo Fisher Scientific) overnight at 4°C. The next day, 50 μl protein A agarose slurry (Pierce) was added to the samples, and the mixture was incubated at room temperature (RT). After 1 h, samples were washed using the lysis buffer. After three washes, the agarose pellet was allowed to dry and subsequently quenched by boiling the samples with SDS buffer at 95°C for 5 min. Samples were loaded on 10% SDS-PAGE gels and analyzed using western blotting.

### Super-resolution imaging

We performed STED super-resolution imaging using a STEDYCON microscope (Abberior). Splenocytes from *depilated* and WT mice were obtained, and immunofluorescence was performed on these cells as previously described elsewhere (Garcia and Boehning, 2017). Briefly, the cells were plated on poly-L-lysine-coated coverslips and treated with anti-CD3 where required. After treatment, the cells were fixed using 4% ice-cold paraformaldehyde for 20 min at room temperature (RT) with rotation. The cells were quenched with 30 mM glycine-PBS solution for 5 min at RT with rotation. Next, the cells were washed three times with PBS with rotation. Cells were permeabilized with 0.25% Triton X-100 and 1% BSA in PBS for 10 min at RT with rotation. Cells were then incubated with 2% BSA in PBS for 1 h at RT with rotation. Cells were washed three times with PBS for 5 min each and then incubated with antibodies (at 1:50) in PBS with 0.3% BSA. Cells were washed and mounted on glass slides sealed with nail polish. The secondary antibodies for STED imaging were Star Red and Star Green anti-rabbit-IgG or anti-mouse-IgG obtained from Abberior Inc and were used at 1:500.

### Fura-2 imaging

Fura-2 imaging on WT HEK293, DHHC21-KO HEK293, and mouse splenocytes was performed as previously described (West et al., 2022b; Kodakandla et al., 2022). Briefly, for adherent cells, 2 days before imaging, cells were seeded on poly-L-lysine-coated coverslips in 6-well tissue culture plates. The next day, these cells were transfected with DHHC21, Orai1 and STIM1 plasmids. Imaging buffer containing 0.1% bovine serum albumin, 107 mM NaCl, 20 mM HEPES, 2.5 mM MgCl_2_, 7.5 mM KCl, 11.5 mM glucose, and 1 mM CaCl_2_, pH 7.2 was used. For store depletion, imaging buffer devoid of CaCl_2_ was used. At 24 h after transfection, the cells were incubated with 5 μM Fura-2 in the imaging buffer for 30 min at RT, followed by de-esterification for 30 min at RT. Time-lapse images were recorded on a Nikon Ti-2 microscope using a 40× oil immersion objective. Cells were excited alternatively at 340 nm and 380 nm every 2 s for 16 min, and the fluorescence emission was collected at 525 nm. The first minute of the time-lapse was used to obtain the baseline Ca^2+^ level. After 1 min, thapsigargin (TG, 10 μM) in the Ca^2+^-free imaging buffer was added to induce store depletion. After an additional 7 min, the buffer was replaced with Ca^2+^-replete imaging buffer with TG to measure Ca^2+^ entry. The ratio of fluorescence at 340 to 380 nm was used to quantify cellular Ca^2+^ levels. Cells that did not respond to TG were excluded from our analysis. Identical exposure parameters were used to obtain images. Peak entry was calculated by subtracting the maximum fluorescence ratio (Rmax) after Ca^2+^ addback from the fluorescence ratio at time 0 (R_0_) and normalized to R_0_.

### Total internal reflection fluorescence imaging

Cells were seeded on poly-L-lysine-coated coverslips 2 days before TIRF imaging. Cells were transfected with WT or ΔF233 DHHC21–eGFP, WT STIM1–mRFP and WT Orai1–Myc using Lipofectamine 3000 following the manufacturer's protocol. A Nikon Eclipse Ti microscope, equipped with a TIRF illumination system, was used for imaging. Coverslips were mounted in Attofluor chambers with the imaging buffer containing Ca^2+^ as used for live imaging above using a 60× oil-immersion TIRF objective. Cells were alternatively excited with 488 nm and 561 nm lasers every 5 s. The first minute of the recording was used to obtain baseline colocalization levels. The images were captured over an 8-min period. After 1 min, the imaging buffer was replaced with 10 μM TG in the Ca^2+^-free imaging buffer. Cells that had puncta prior to store depletion or those that showed high colocalization in the resting state were excluded from analysis. Colocalization analysis was performed using Nikon NIS Elements software. Using regions of interest around cells, we obtained Pearson's correlation coefficients between both channels for the entire time series. We normalized correlation coefficients to time zero by dividing the correlation coefficient at a given time (R_n_) by time zero (R_0_). These values were used to plot the colocalization time curve. Peak colocalization was calculated by subtracting peak colocalization after TG addition (R_max_) from colocalization just before TG addition (R_60_) and dividing the difference by R_60_ [(R_max_−R_60_)/R_60_].

### Confocal imaging

DHHC21-KO cells plated on coverslips and transfected with WT and ΔF233 versions of DHHC21–eGFP were used for confocal imaging. The cells were imaged using the confocal mode on the STEDYCON microscope described above, using a 100× oil objective. Identical exposure parameters were used to obtain images from WT and ΔF233 DHHC21-expressing cells.

### Caspase-3 activity assay

Splenocytes from WT or homozygous *Zdhhc21*^dep/dep^ mice were treated with JO-2 for 24 h. Total caspase activity was determined in cell lysates using the caspase-3 substrate Z-DEVD-R110 as described previously (Wozniak et al., 2006). The production of fluorescent substrate was monitored continuously every minute for 1 h in a microplate reader. The slope of the linear regression drawn through each time point was used to determine the change in fluorescence over time for each sample.

### ELISA

ELISA tests for ANA, CRP, and VB12 were purchased from Thermo Fisher Scientific (501487635, EM20RB, 502287196). Serum was collected from 6–8-week-old mice. The total amounts of each analyte were determined as per the manufacturer's instructions.

### Software

All microscopy data were acquired using Nikon Elements, except for STED images, which were acquired via the Abberior STEDYCON browser-based interface. Data analysis was performed in ImageJ or Excel, and data were plotted in GraphPad Prism 11. Statistical tests were performed in GraphPad Prism 11.

## Supplementary Material



10.1242/joces.264681_sup1 Supplementary information
