# Supplementary material for: DHHC21 is a STIM1 protein S-acyltransferase that modulates immune function in vivo
Source: J Cell Sci. 2026 Sep 11;139(17):jcs264681. doi: 10.1242/jcs.264681 (PMC13615677; doi:10.1242/jcs.264681)
Supplement: Supplementary file 1 [file joces-139-264681-s1.pdf]

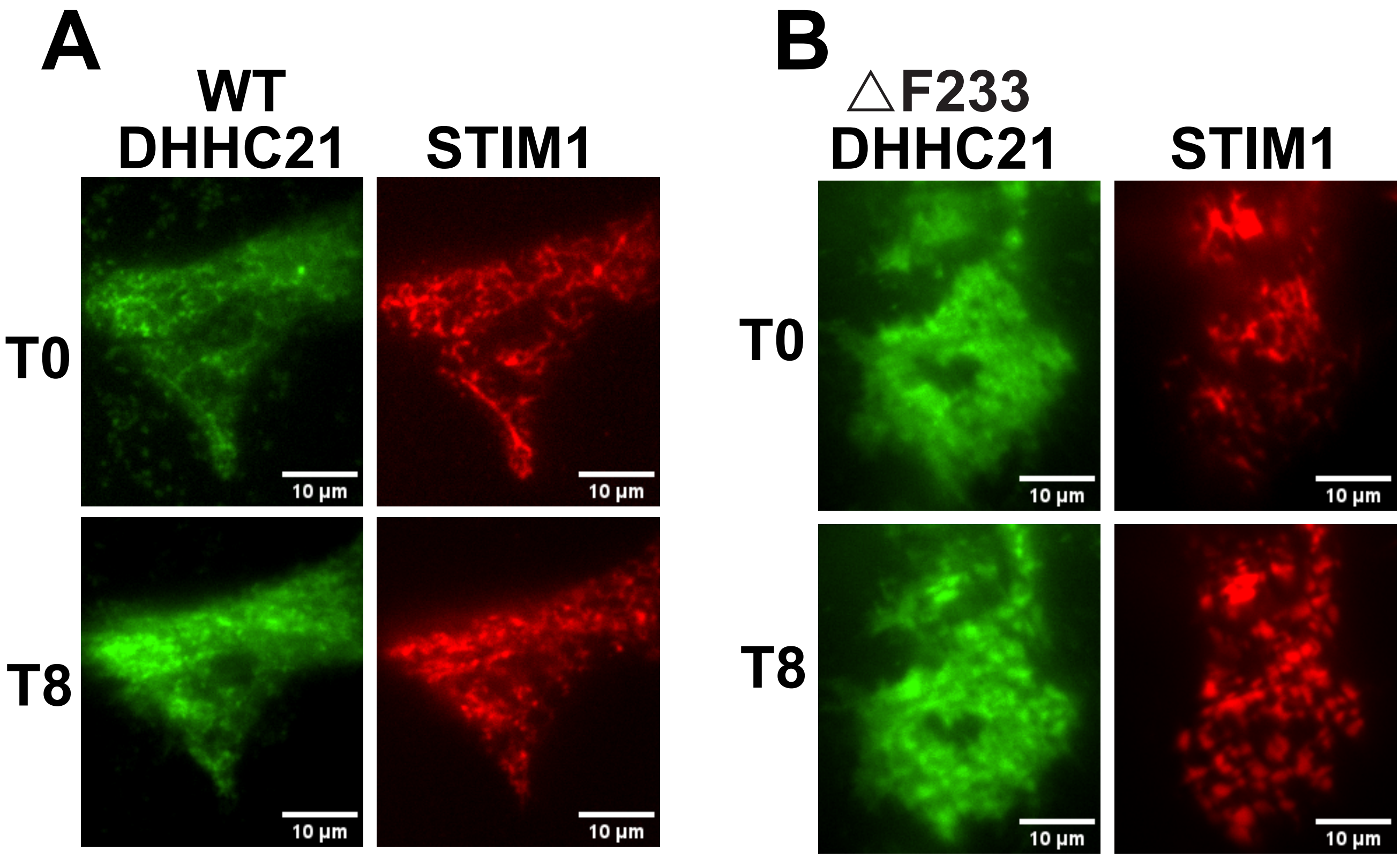

Fig. S1. Individual channels from Fig. 3B. A) WT DHHC21 (green) and STIM1 (red) at time zero (T0) and eight minutes after TG treatment (T8). B) ΔF233 DHHC21 (green) and STIM1 (red) at time zero (T0) and eight minutes after TG treatment (T8).

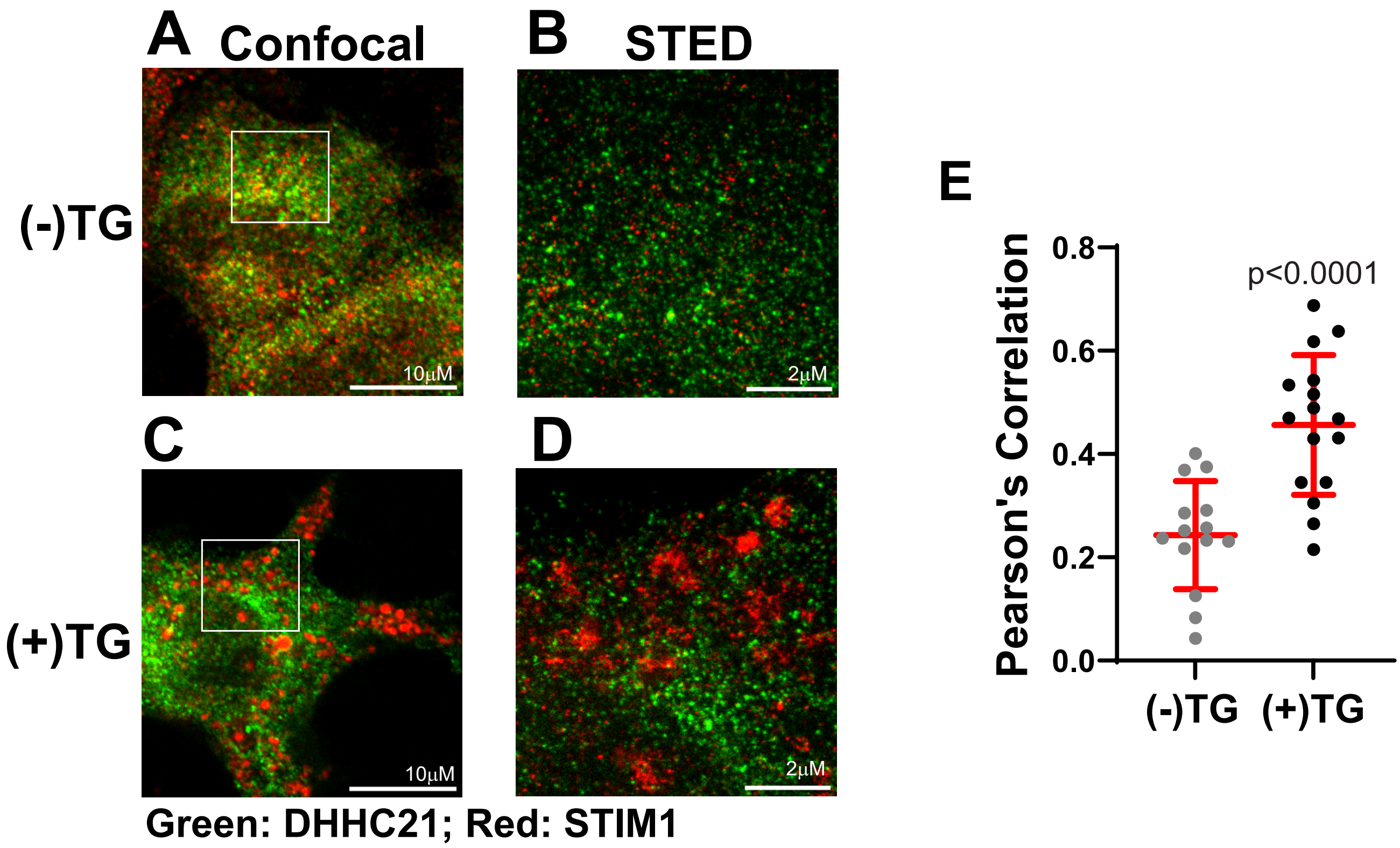

Fig. S2. Endogenous DHHC21 colocalizes with STIM1 in HEK293 WT cells. Confocal (A and C) and STED (B and C) images of HEK293 WT cells untreated (A and B) or treated (C and D) with 10 μM thapsigargin (TG). The boxes in Panels A and C were used as the imaging area for STED imaging in Panels B and D. Green represents DHHC21, and red represents STIM1. E) Colocalization was quantified using Pearson's correlation values obtained from cells. Statistical significance between the groups was calculated using Student's t-test. Error bars indicate S.D. Pearson's correlation between DHHC21 and STIM1 obtained from (E) using the JACoP plugin was analyzed using a Student's t-test. Error bars indicate S.D. Total number of cells analyzed were as follows 14 (-TG) and 16 (+TG).

Journal of Cell Science • Supplementary information

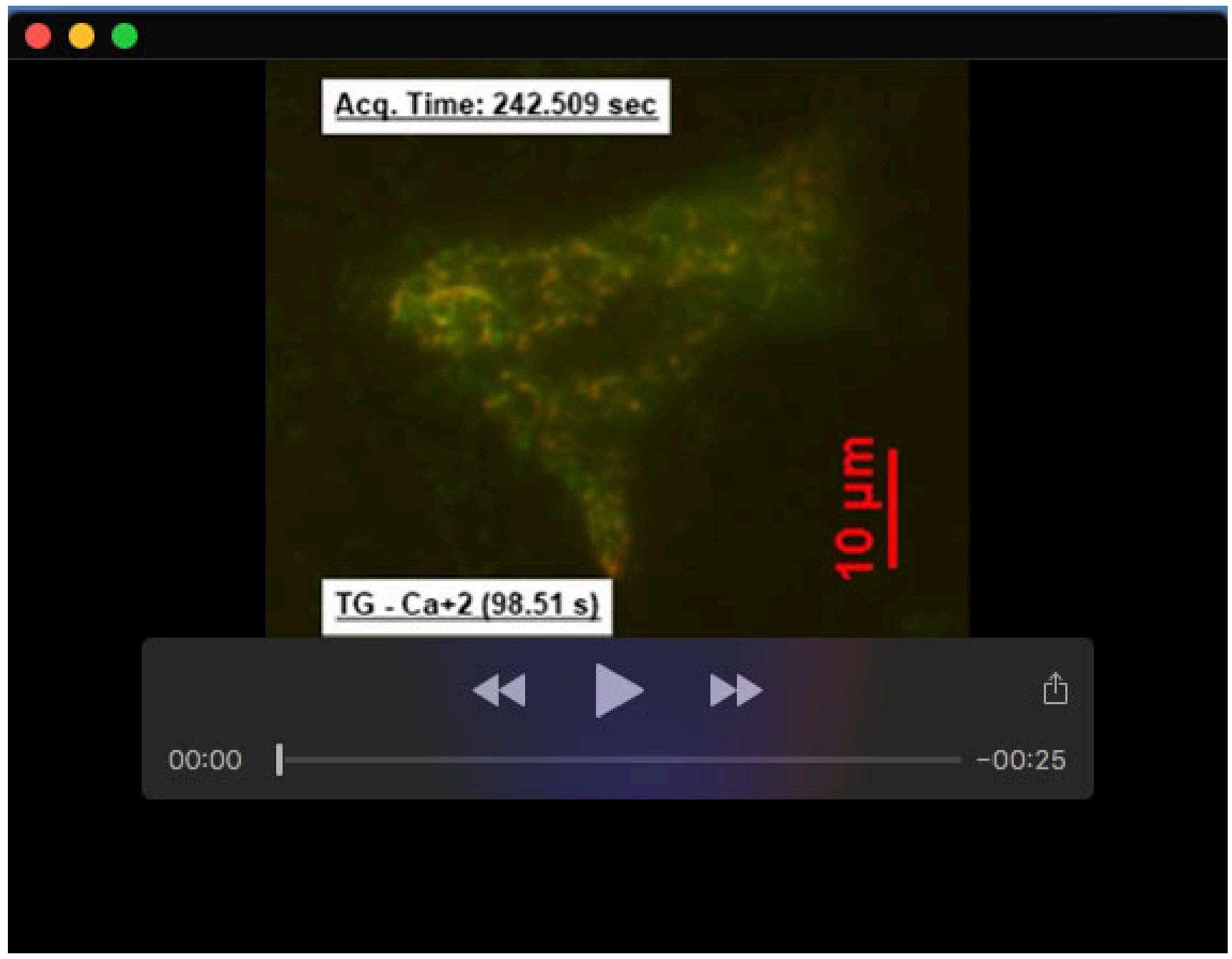

Movie 1. DHHC21 and STIM1 colocalize upon ER store depletion. DHHC21 KO cells were transfected with DHHC21-GFP and STIM1-mRFP, and TIRF microscopy was performed. Store depletion was induced using 10 µM thapsigargin (TG).

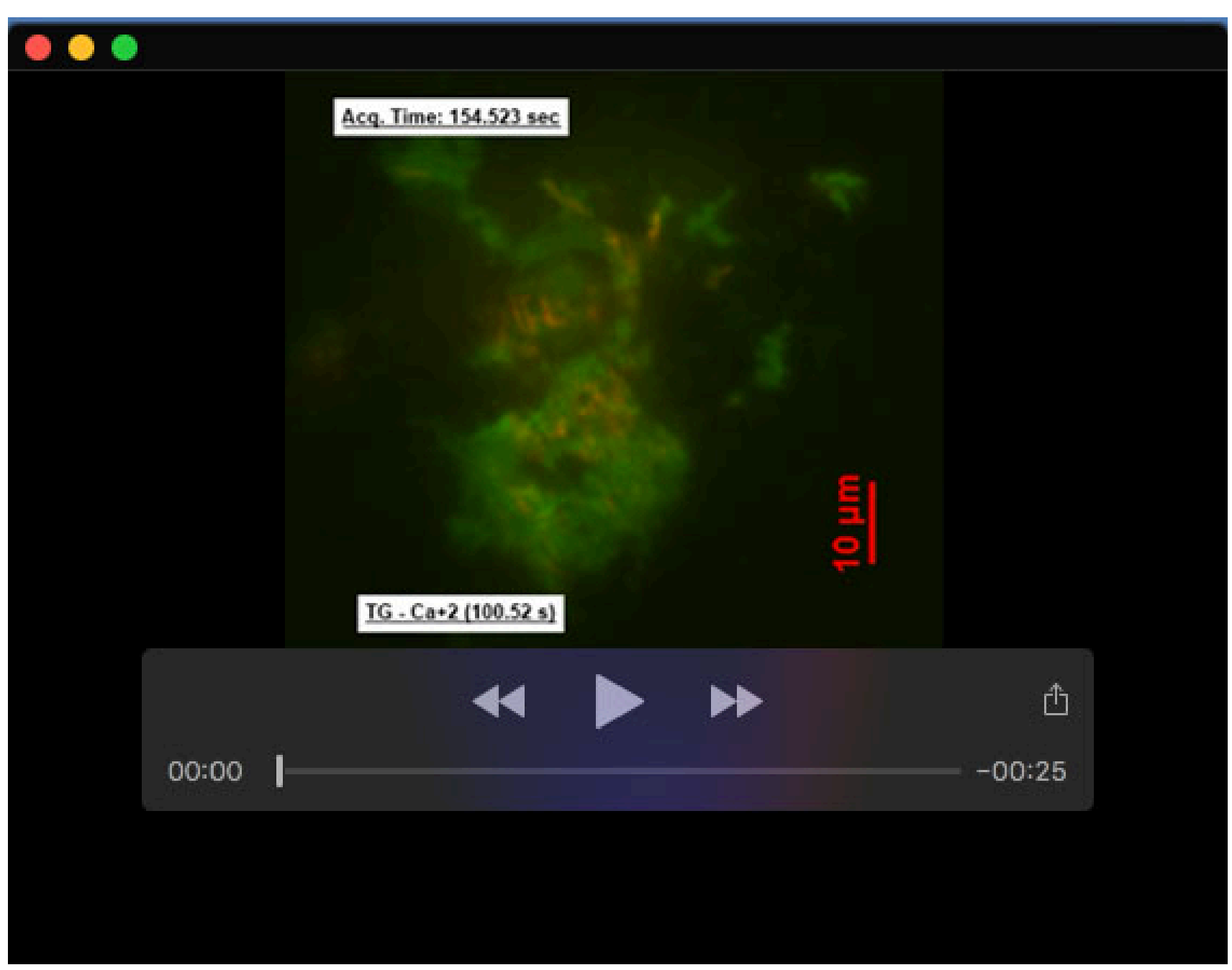

Movie 2. ΔF233 DHHC21 and STIM1 colocalize upon ER store depletion. DHHC21 KO cells were transfected with ΔF233 DHHC21-GFP and STIM1-mRFP, and TIRF microscopy was performed. Store depletion was induced using 10 µM thapsigargin (TG).
