# Supplementary material for: DHHC21 is a STIM1 protein S-acyltransferase that modulates immune function in vivo
Source: J Cell Sci. 2026 Sep 11;139(17):jcs264681. doi: 10.1242/jcs.264681 (PMC13615677; doi:10.1242/jcs.264681)
Supplement: Supplementary file 2 [file joces-139-264681_review_history.pdf]

DHHC21 is a STIM1 protein S-acyltransferase that modulates immune function *in vivo*

Goutham Kodakandla, Ying Fan, Michael X. Zhu, Savannah J. West, Askar Akimzhanov and Darren Boehning

DOI: 10.1242/jcs.264681

Editor: Sophie Acton

Review timeline

Original submission:	22 December 2025
Editorial decision:	21 January 2026
First revision received:	15 May 2026
Editorial decision:	25 June 2026
Second revision received:	28 July 2026
Accepted:	4 August 2026

Original submission

First decision letter

MS ID#: jcs.264681

MS TITLE: DHHC21 is a STIM1 protein S-acyltransferase that modulates immune function in vivo

AUTHORS: Goutham Kodakandla; Ying Fan; Michael Zhu; Savannah West; Askar Akimzhanov; Darren Boehning

ARTICLE TYPE: Transfer

Dear Dr Boehning,

We have now reached a decision on the above manuscript.

As you will see, the reviewers raise a number of substantial criticisms that prevent me from accepting the paper at this stage. They suggest, however, that a revised version might prove acceptable, if you can address their concerns. If you think that you can deal satisfactorily with the criticisms on revision, I would be pleased to see a revised manuscript. We would then return it to the reviewers.

Reviewer 1

SUMMARY OF THE ADVANCE MADE IN THIS PAPER AND ITS POTENTIAL SIGNIFICANCE TO THE FIELD

This manuscript identifies the protein S-acyltransferase DHHC21 as a regulator of STIM1 S-acylation and store-operated calcium entry (SOCE), and links DHHC21 dysfunction to immune abnormalities resembling autoimmune lymphoproliferative syndrome (ALPS). Using a combination of mouse genetics, biochemical assays, calcium assays, and advanced microscopy, the authors propose that DHHC21 is the principal palmitoyl acyltransferase for STIM1 in vivo.

© 2026. Published by The Company of Biologists under the terms of the Creative Commons Attribution License (https://creativecommons.org/licenses/by/4.0/).

The study addresses an important and timely question in calcium signalling and immune regulation. The in vivo data using the depilated mouse model are a notable strength, and the mechanistic link between DHHC21, STIM1, and SOCE is generally well supported. However, several key issues regarding specificity, interpretation of the ΔF233 mutation, and the extent of ALPS phenotyping need to be addressed before the manuscript is suitable for publication.

SUGGESTIONS TO AUTHORS

Major comments [Please request additional experiments only if they are essential for supporting the conclusions; authors should be encouraged to highlight any claims that are preliminary or speculative, or to discuss any pitfalls or alternative interpretations in a 'Limitations' section]

1. Specificity of DHHC21 as the STIM1 acyltransferase

The authors conclude that DHHC21 is the primary palmitoyl acyltransferase (PAT) for STIM1. While the data clearly demonstrate a major role for DHHC21, this claim is not fully substantiated.

Other plasma membrane-localized DHHC enzymes (e.g. DHHC5 and DHHC20) are discussed in the introduction but not experimentally tested.

Residual SOCE in depilated splenocytes and in DHHC21-knockout HEK293 cells suggests partial redundancy or compensation.

Suggested revisions:

Test whether knockdown of other PM-localized DHHCs further impairs SOCE in the DHHC21 KO background, or

Assess whether overexpression of DHHC5 or DHHC20 can rescue STIM1 S-acylation or SOCE.

At minimum, the authors should moderate the claim of DHHC21 being the "primary" PAT for STIM1.

2. The ΔF233 mutation may not be a simple loss-of-function This is acknowledged by the authors in the discussion (lines 214-230), but it creates interpretive challenges throughout the manuscript. The comparison with the recent T-cell-specific DHHC21 knockout study (Meng et al., 2025) showing opposite phenotypes (smaller spleens vs. splenomegaly) is concerning.
Suggestions:

The authors should more clearly distinguish which phenotypes can be attributed to loss of STIM1 S-acylation versus potential gain-of-function effects of substrate sequestration

Minor comments
Baseline calcium differences: The elevated baseline calcium in depilated splenocytes (Figure 1E) and ΔF233-rescued cells (Figure 2F) is interesting but not mechanistically explained. Does this reflect altered ER calcium content, plasma membrane calcium leak, or altered PMCA/NCX activity?
TG-releasable pool: The reduced TG-releasable pool in depilated splenocytes (Figure 1G) but not in ΔF233-rescued DHHC21 KO cells (Figure 2G) is discrepant. This should be discussed.
Superresolution imaging quantification: For Figure 3E-F, how many cells were analyzed per condition? The colocalization values show high variability.

Reviewer 2

SUMMARY OF THE ADVANCE MADE IN THIS PAPER AND ITS POTENTIAL SIGNIFICANCE TO THE FIELD

In this manuscript titled, "***DHHC21 is a STIM1 protein S-acyltransferase that modulates immune function in vivo***", the authors show that DHHC21 is essential for STIM1 S-acylation and subsequent store-operated Ca^{2+} entry. They also demonstrate DHHC21 physically interacts and co-localize with STIM1 upon store depletion. Moreover, animal model harboring DHHC21 mutation recapitulate features of autoimmune lymphoproliferative syndrome. Overall, these results from the leaders in this field are compelling. My comments and suggestions are mentioned below:

© 2026. Published by The Company of Biologists under the terms of the Creative Commons Attribution License (https://creativecommons.org/licenses/by/4.0/).

SUGGESTIONS TO AUTHORS

Major comments

1. Please include a positive control with differential S-acylation in the acyl-RAC assay (example, components of Fas or TCR complexes).
2. It is surprising that over-expression of WT DHHC21 did not affect S-acylation. Does this mean that STIM1 acylation is saturated at baseline? What is the effect of over-expressing WT DHHC21 on SOCE?
3. Can the higher resting baseline Ca2+ levels be restored to WT levels using an Orai inhibitor in depilated splenocytes or possibly in rescue experiments with DHHC21 ΔF233 in DHHC21 KO cells?
4. The traces and numbers in the pooled data (2D-G) in rescue experiments with WT and DHHC21 ΔF233 (compared to 2B-C) are compounded by the expression of DHHC21-eGFP followed by Fura assays. Thus, SOCE was completely restored in WT DHHC21 expressing DHHC21 KO cells is an overstatement.
5. Co-IP assays lack proper controls such as isotype (IgG), bead-only control. Can you please verify the details of antibodies employed in Co-IP in the materials and methods section?

Minor comments

1. Figure 1 legend. Since S-acylation of calnexin was not altered, it can't serve as positive control.
2. Figure 2A legend. Calnexin or GAPDH?
3. Figure 2B and C. Peak from the representative look similar. Will calculating the area under the curve be more appropriate in this case?

First revision

<u>Author response to reviewers' comments</u>

Dear Dr. Acton and Reviewers,

We would like to thank the reviewers for their constructive comments, which we feel have greatly improved this manuscript. Below, we provide a point-by-point discussion of each of the points raised by the reviewers. We hope that the manuscript is now suitable for publication.

REVIEWER 1

1. Specificity of DHHC21 as the STIM1 acyltransferase. The authors conclude that DHHC21 is the primary palmitoyl acyltransferase (PAT) for STIM1. While the data clearly demonstrate a major role for DHHC21, this claim is not fully substantiated. Other plasma membrane-localized DHHC enzymes (e.g. DHHC5 and DHHC20) are discussed in the introduction but not experimentally tested. Residual SOCE in depilated splenocytes and in DHHC21-knockout HEK293 cells suggests partial redundancy or compensation.

We thank the reviewer for this excellent suggestion, which has significantly added to our findings. Using DHHC20 siRNA-mediated knockdown in DHHC21 CRISPR KO cells, we now show that DHHC20 KD leads to a reduction in the residual SOCE. We also performed Acyl-RAC on WT, KO, and KO+KD cells, and showed that DHHC20 KD further reduces STIM1 S-acylation in DHHC21 KO cells. Interestingly, DHHC20 over expression does not rescue SOCE in KO cells. We interpret this in the paper as follows: *"The DHHC20 enzyme also regulates STIM1 S-acylation, but cannot rescue SOCE in DHHC21 KO cells. This may represent a distinct role in STIM1 regulation, or alternatively, that overexpressed DHHC20 does not recapitulate the function of the endogenous enzyme."* We have changed the language throughout the entire manuscript to reflect this new data and acknowledge the potential role of DHHC20.

© 2026. Published by The Company of Biologists under the terms of the Creative Commons Attribution License (https://creativecommons.org/licenses/by/4.0/).

2. The ΔF233 mutation may not be a simple loss-of-function. This is acknowledged by the authors in the discussion (lines 214-230), but it creates interpretive challenges throughout the manuscript. The comparison with the recent T-cell-specific DHHC21 knockout study (Meng et al., 2025) showing opposite phenotypes (smaller spleens vs. splenomegaly) is concerning.
Suggestions:
The authors should more clearly distinguish which phenotypes can be attributed to loss of STIM1 S-acylation versus potential gain-of-function effects of substrate sequestration

We agree that the ΔF233 mutation poses many interpretive challenges. Even the DHHC21 CRISPR KO (absent ΔF233) can be difficult to interpret, as DHHC21 is a promiscuous enzyme with many substrates (indeed, we have identified a large number of them in T cells in separate publications). Direct versus indirect effects are difficult to discern. A few straightforward observations:
1. Eliminating the endogenous DHHC21 gene in cultured cells causes a large loss in SOCE and STIM1 S-acylation. The ΔF233 mutation cannot fully rescue this phenotype.
2. DHHC21 binds to STIM1 and is actively recruited into puncta during store depletion.
3. Splenocytes from *depilated* mice have a phenotype similar (but not identical) to HEK cells with KO of DHHC21.
The DHHC21 ΔF233 mutation does not affect the active site, but rather eliminates its regulation by calmodulin. We provided one possible interpretation (substrate sequestration), but it could also be that the enzyme is still functional (and S-acylates substrates), but is no longer activated in a stimulus-dependent manner. This would be expected to have a different phenotype *in vivo* versus a knockout. We have provided a more concise summary of this statement in the discussion section of the paper.

Minor comments
Baseline calcium differences: The elevated baseline calcium in depilated splenocytes (Figure 1E) and ΔF233-rescued cells (Figure 2F) is interesting but not mechanistically explained. Does this reflect altered ER calcium content, plasma membrane calcium leak, or altered PMCA/NCX activity?

We have added text suggesting that the increased cytosolic calcium in splenocytes is due to altered SERCA or IP3R activity. This is based upon the finding that ER stores are depleted in depilated splenocytes (Figure 1G). Interestingly, we recapitulated this finding (higher cytosolic calcium) in HEK cells by expressing ΔF233 (Figure 2G), but store size was not affected in vitro (as noted by the reviewer in the next comment). Future work will be needed to evaluate this hypothesis in more detail.

TG-releasable pool: The reduced TG-releasable pool in depilated splenocytes (Figure 1G) but not in ΔF233-rescued DHHC21 KO cells (Figure 2G) is discrepant. This should be discussed.

We have added text to the description of Figure 2H (formerly 2G) in the discussion.

Superresolution imaging quantification: For Figure 3E-F, how many cells were analyzed per condition? The colocalization values show high variability.

We have added the total number of cells imaged to the Figure Legend.

REVIEWER 2

1. Please include a positive control with differential S-acylation in the acyl-RAC assay (example, components of Fas or TCR complexes).

Thank you for this comment. None of Acyl-RAC experiments in this manuscript were done after cellular stimulation. In other words, they are all done at baseline/steady state. We have always used calnexin (CLXN) S-acylation as a loading control since it is a stably S-acylated protein (1-5). We conclude that both in spleen depilated lysates (Figure 1A) and DHHC21 KO cells (Figure 2), STIM1 is a substrate of DHHC21.

© 2026. Published by The Company of Biologists under the terms of the Creative Commons Attribution License (https://creativecommons.org/licenses/by/4.0/).

2. It is surprising that over-expression of WT DHHC21 did not affect S-acylation. Does this mean that STIM1 acylation is saturated at baseline? What is the effect of over-expressing WT DHHC21 on SOCE?

As requested, we examined SOCE in DHHC21-overexpressing HEK cells (Figure 2C) and observed no increase in TG-induced calcium entry. We know the enzyme is functional, as we can at least partially rescue SOCE in DHHC21 KO cells (Figure 2F). This is hand waving, but it is possible that the cell is regulating the amount of active DHHC21 at the plasma membrane to maintain cellular homeostasis. We do know that the recombinant DHHC21 protein reaches the plasma membrane and can be incorporated into puncta (Figure 3B-D). The strength of our data relies on the single amino acid substitution of the endogenous gene (ΔF233; in vivo) and CRISPR KO of the gene (in vitro) models rather than overexpression models.

3. Can the higher resting baseline Ca2+ levels be restored to WT levels using an Orai inhibitor in depilated splenocytes or possibly in rescue experiments with DHHC21 ΔF233 in DHHC21 KO cells?

We show in Figure 2G that rescuing ΔF233 expression in DHHC21 KO cells increases cytosolic calcium levels. As noted in a comment for reviewer #1, we have added text suggesting that the increased cytosolic calcium in splenocytes is due to altered SERCA or IP3R activity. This is based upon the finding that ER stores are depleted in depilated splenocytes (Figure 1G). Somewhat surprisingly, store size was not affected by ΔF233 expression in DHHC21 KO cells. This may suggest distinct calcium-handling defects in each model. We have noted this in the text of the manuscript.

4. The traces and numbers in the pooled data (2D-G) in rescue experiments with WT and DHHC21 ΔF233 (compared to 2B-C) are compounded by the expression of DHHC21-eGFP followed by Fura assays. Thus, SOCE was completely restored in WT DHHC21 expressing DHHC21 KO cells is an overstatement.

We have greatly softened our interpretations throughout the manuscript, noting *partial* rescue and including new data indicating that DHHC20 may also contribute to STIM1 S-acylation (see the comments to reviewer #1).

5. Co-IP assays lack proper controls such as isotype (IgG), bead-only control. Can you please verify the details of antibodies employed in Co-IP in the materials and methods section?

We have provided a new Figure 3A of much higher quality, and the inclusion of beads-only controls. We have updated the methods to clearly note the antibodies used.

Minor comments
1. Figure 1 legend. Since S-acylation of calnexin was not altered, it can't serve as positive control.

We have changed the wording to state “loading control” exclusively. See the comment above RE dynamic S-acylated substrates.

2. Figure 2A legend. Calnexin or GAPDH?

We apologize for this mistake. GAPDH is the correct loading control. We use calnexin as a loading control for Acyl-RAC.

3. Figure 2B and C. Peak from the representative look similar. Will calculating the area under the curve be more appropriate in this case?

The Peak TG release is indeed similar and is representative of store size. The peak entry is significantly lower in both the representative trace (2B) and the quantified data (2C). As noted in the discussion points above, the HEK KO model does not affect store size like the *depilated* splenocytes.

We would like to sincerely thank the reviewers, who have truly improved this manuscript. We hope that it is now suitable for publication.

© 2026. Published by The Company of Biologists under the terms of the Creative Commons Attribution License (https://creativecommons.org/licenses/by/4.0/).

References

1. Akimzhanov, A. M., and Boehning, D. (2015) Rapid and transient palmitoylation of the tyrosine kinase Lck mediates Fas signaling Proc Natl Acad Sci U S A **112**, 11876-11880 10.1073/pnas.1509929112
2. Chen, J. J., Marsden, A. N., Scott, C. A., Akimzhanov, A. M., and Boehning, D. (2020) DHHC5 Mediates beta-Adrenergic Signaling in Cardiomyocytes by Targeting Galpha Proteins Biophys J **118**, 826-835 10.1016/j.bpj.2019.08.018
3. Bieerkehazhi, S., Fan, Y., West, S. J., Tewari, R., Ko, J., Mills, T. *et al.* (2022) Ca2+-dependent protein acyltransferase DHHC21 controls activation of CD4+ T cells J Cell Sci **135**, 10.1242/jcs.258186
4. Kodakandla, G., West, S. J., Wang, Q., Tewari, R., Zhu, M. X., Akimzhanov, A. M. *et al.* (2022) Dynamic S-acylation of the ER-resident protein stromal interaction molecule 1 (STIM1) is required for store-operated Ca(2+) entry J Biol Chem **298**, 102303 10.1016/j.jbc.2022.102303
5. West, S. J., Kodakandla, G., Wang, Q., Tewari, R., Zhu, M. X., Boehning, D. *et al.* (2022) S-acylation of Orai1 regulates store-operated Ca2+ entry J Cell Sci **135**, 10.1242/jcs.258579

Second decision letter

MS ID#: jcs.264681R1

MS TITLE: DHHC21 is a STIM1 protein S-acyltransferase that modulates immune function in vivo

AUTHORS: Goutham Kodakandla; Ying Fan; Michael Zhu; Savannah West; Askar Akimzhanov; Darren Boehning

ARTICLE TYPE: Transfer

Dear Dr Boehning,

We have now reached a decision on the above manuscript.

As you will see, the reviewers gave favourable reports but raised a few minor points that will require amendments to your manuscript. I hope that you will be able to carry these out because I would like to be able to accept your paper, depending on further comments from reviewers.

Reviewer 1

SUMMARY OF THE ADVANCE MADE IN THIS PAPER AND ITS POTENTIAL SIGNIFICANCE TO THE FIELD

Comments to the Authors
I thank the authors for a thorough and good-faith revision. The new DHHC20 knockdown and Acyl-RAC experiments meaningfully strengthen the manuscript, and the reframing of the depilated phenotype in light of Meng et al. (2025) is a thoughtful way to address the loss- versus gain-of-function question raised in the previous review.
My remaining comments are intended to help the authors present their conclusions as clearly and defensibly as possible. None of these points require new in vivo work, and most relate to wording, consistency, statistical reporting, and correction of one figure legend.
Major point
1. Calibrate the central claim to the new data.
The DHHC20 experiments are a valuable addition. However, they now sit somewhat awkwardly with the Discussion statement that "we show that DHHC21 is the PAT that S-acylates STIM1 in vivo." Having shown that DHHC20 knockdown further reduces STIM1 S-acylation and SOCE, the phrase "the PAT" is stronger than the data now support. The Abstract's "major mediator" framing is more appropriate and more defensible. I recommend bringing the Discussion, and the related

© 2026. Published by The Company of Biologists under the terms of the Creative Commons Attribution License (https://creativecommons.org/licenses/by/4.0/).

Introduction statement that "we examined the hypothesis that DHHC21 is the PAT for STIM1," into alignment with this more calibrated wording.
For example, the authors could refer to DHHC21 as "a major STIM1 S-acyltransferase" or "a central mediator of STIM1 S-acylation and SOCE," rather than "the PAT." This would strengthen rather than weaken the manuscript, because it better reflects the newly added redundancy data.
Relatedly, my original comment mentioned both DHHC5 and DHHC20. DHHC20 has now been pursued thoroughly, but a single sentence noting why DHHC5 was not tested would close that loop. For instance, the authors could briefly mention DHHC5's distinct substrate profile, if that is their rationale.
Minor points
1. Figure 2 legend does not match the revised figure or the main text.
Figure 2 now contains eight panels, A-H: A, Western blot; B, DHHC21 KO traces; C, peak calcium entry across five conditions; D, the new Acyl-RAC blot; E, rescue traces; F, peak calcium entry; G, baseline calcium; and H, TG-releasable pool.
However, the legend still describes "D-G" as Fura-2 imaging, with traces in D, peak entry in E, baseline calcium in F, and TG-releasable pool in G. This omits the new Acyl-RAC panel and shifts every subsequent panel by one. The main text appears to cite the panels correctly, including Fig. 2D for Acyl-RAC and Fig. 2F-H for the quantifications, so only the legend needs to be revised. I flag this specifically because panel D was added in response to review, and it is therefore particularly important that the legend accurately describes it.
2. Text slip in the same section.
The sentence "We found that DHHC21 S-acylation was reduced in KO cells" should read "STIM1 S-acylation was reduced in KO cells." One cannot measure DHHC21's own acylation in a DHHC21 knockout, and the panel appears to blot STIM1.
3. Statistical test and sample-size reporting for Figure 2C.
Figure 2C compares five conditions: HEK WT, DHHC21 KO, DHHC21 overexpression, DHHC21 KO + DHHC20 knockdown, and DHHC21 KO + DHHC20 overexpression. The panel displays multiple p-values, but the legend specifies Student's t-test. A one-way ANOVA with appropriate post-hoc correction would be more suitable for this comparison. The authors already use this approach in Figure 3F, so this should be a consistency correction rather than a new analytical framework.
In addition, the Figure 2C legend reports n values for only two of the five groups, namely 108 WT and 117 KO cells. Please add the n values for the remaining three groups.
Separately, Figure 4H presents four groups — WT and depilated mice, each split into <9 months and >9 months — under a single p-value. It would help to state explicitly which comparison that p-value refers to. Figure 4A's two age-bracketed WT-versus-depilated comparisons read as appropriate as currently presented.
4. Reference duplication.
Carreras-Sureda et al. 2021a and Carreras-Sureda et al. 2021b appear to be the same paper, with identical authors, title, journal, and volume. They differ only in the formatting of "Ca2+" versus "Ca(2+)" in the title. These entries are cited under both labels in different parts of the manuscript. Please merge them into a single reference entry and reconcile the in-text citations accordingly.

<u>Reviewer 2</u>

1. With respect to my previous comment 4. *“The traces and numbers in the pooled data (2D-G) in rescue experiments with WT and DHHC21 ΔF233 (compared to 2B-C) are compounded by the expression of DHHC21-eGFP followed by Fura assays. Thus, SOCE was completely restored in WT DHHC21 expressing DHHC21 KO cells is an overstatement”*, I agree, in the revised version the authors softened their interpretations. Yet my concern is that GFP fluorescence might interfere with Fura/Ca2+ measurements. In these experiments, are both the wild-type and ΔF233 DHHC21 tagged with eGFP? If yes, please explicitly mention this in the manuscript.

2. The authors revised Fig. 3A to include bead-only control. That’s nice (partially addressed). I was also hoping to see an IgG control as an **essential negative control** used to differentiate true, specific protein-protein interactions from false-positive background noise. It verifies that the proteins pulled down are bound by your target antibody, not the antibody itself. Not mandatory now but just a suggestion for future Co-Ips.

© 2026. Published by The Company of Biologists under the terms of the Creative Commons Attribution License (https://creativecommons.org/licenses/by/4.0/).

Second revision

Author response to reviewers' comments

Dear Dr. Acton and Reviewers,

We would like to thank the reviewers for their constructive comments, which we feel have greatly improved this manuscript. Below, we provide a point-by-point discussion of each of the points raised by the reviewers. We hope that the manuscript is now suitable for publication.

REVIEWER 1
I thank the authors for a thorough and good-faith revision. The new DHHC20 knockdown and Acyl-RAC experiments meaningfully strengthen the manuscript, and the reframing of the depilated phenotype in light of Meng et al. (2025) is a thoughtful way to address the loss- versus gain-of-function question raised in the previous review. My remaining comments are intended to help the authors present their conclusions as clearly and defensibly as possible. None of these points require new in vivo work, and most relate to wording, consistency, statistical reporting, and correction of one figure legend.

Major point
1. Calibrate the central claim to the new data.
The DHHC20 experiments are a valuable addition. However, they now sit somewhat awkwardly with the Discussion statement that "we show that DHHC21 is the PAT that S-acylates STIM1 in vivo." Having shown that DHHC20 knockdown further reduces STIM1 S-acylation and SOCE, the phrase "the PAT" is stronger than the data now support. The Abstract's "major mediator" framing is more appropriate and more defensible. I recommend bringing the Discussion, and the related Introduction statement that "we examined the hypothesis that DHHC21 is the PAT for STIM1," into alignment with this more calibrated wording.
For example, the authors could refer to DHHC21 as "a major STIM1 S-acyltransferase" or "a central mediator of STIM1 S-acylation and SOCE," rather than "the PAT." This would strengthen rather than weaken the manuscript, because it better reflects the newly added redundancy data.
Relatedly, my original comment mentioned both DHHC5 and DHHC20. DHHC20 has now been pursued thoroughly, but a single sentence noting why DHHC5 was not tested would close that loop. For instance, the authors could briefly mention DHHC5's distinct substrate profile, if that is their rationale.

We thank the reviewers for this input. We have changed the language to a major PAT. We also added some clarification for DHHC5's role.

Minor points
1. Figure 2 legend does not match the revised figure or the main text.
Figure 2 now contains eight panels, A-H: A, Western blot; B, DHHC21 KO traces; C, peak calcium entry across five conditions; D, the new Acyl-RAC blot; E, rescue traces; F, peak calcium entry; G, baseline calcium; and H, TG-releasable pool. However, the legend still describes "D-G" as Fura-2 imaging, with traces in D, peak entry in E, baseline calcium in F, and TG-releasable pool in G. This omits the new Acyl-RAC panel and shifts every subsequent panel by one. The main text appears to cite the panels correctly, including Fig. 2D for Acyl-RAC and Fig. 2F-H for the quantifications, so only the legend needs to be revised. I flag this specifically because panel D was added in response to review, and it is therefore particularly important that the legend accurately describes it.

We have updated the figure legend.

2. Text slip in the same section.
The sentence "We found that DHHC21 S-acylation was reduced in KO cells" should read "STIM1 S-acylation was reduced in KO cells." One cannot measure DHHC21's own acylation in a DHHC21 knockout, and the panel appears to blot STIM1.

We have corrected this discrepancy.

© 2026. Published by The Company of Biologists under the terms of the Creative Commons Attribution License (https://creativecommons.org/licenses/by/4.0/).

3. Statistical test and sample-size reporting for Figure 2C.
Figure 2C compares five conditions: HEK WT, DHHC21 KO, DHHC21 overexpression, DHHC21 KO + DHHC20 knockdown, and DHHC21 KO + DHHC20 overexpression. The panel displays multiple p-values, but the legend specifies Student's t-test. A one-way ANOVA with appropriate post-hoc correction would be more suitable for this comparison. The authors already use this approach in Figure 3F, so this should be a consistency correction rather than a new analytical framework. In addition, the Figure 2C legend reports n values for only two of the five groups, namely 108 WT and 117 KO cells. Please add the n values for the remaining three groups.

We apologize for the confusion. When we added additional conditions during review, we performed a one-way ANOVA followed by Tukey post-hoc analysis. The reference to a t-test was inadvertently left over from the previous submission. We have corrected this in the legend. We also added the n values for the panel.

Separately, Figure 4H presents four groups — WT and depilated mice, each split into <9 months and >9 months — under a single p-value. It would help to state explicitly which comparison that p-value refers to. Figure 4A's two age-bracketed WT-versus-depilated comparisons read as appropriate as currently presented.

We have added the clarification for analysis for Fig 4H. Age was the independent variable in that analysis.

4. Reference duplication.
Carreras-Sureda et al. 2021a and Carreras-Sureda et al. 2021b appear to be the same paper, with identical authors, title, journal, and volume. They differ only in the formatting of "Ca2+" versus "Ca(2+)" in the title. These entries are cited under both labels in different parts of the manuscript. Please merge them into a single reference entry and reconcile the in-text citations accordingly.

We have corrected the duplication. It reads as a single reference now.

REVIEWER 2
1. With respect to my previous comment 4. "The traces and numbers in the pooled data (2D-G) in rescue experiments with WT and DHHC21 ΔF233 (compared to 2B-C) are compounded by the expression of DHHC21-eGFP followed by Fura assays. Thus, SOCE was completely restored in WT DHHC21 expressing DHHC21 KO cells is an overstatement", I agree, in the revised version the authors softened their interpretations. Yet my concern is that GFP fluorescence might interfere with Fura/Ca2+ measurements. In these experiments, are both the wild-type and ΔF233 DHHC21 tagged with eGFP? If yes, please explicitly mention this in the manuscript.

Fura2 has a distinct excitation profile (340nm/380nm) compared to eGFP (488nm). While autofluorescence due to UV excitation might affect eGFP fluorescence, it will affect both WT and ΔF233 similarly. We routinely measure eGFP and YFP with Fura-2 in calcium imaging experiments and have published extensively using this approach. We have added the eGFP descriptions for WT and ΔF233 to the manuscript, as the reviewer suggested.

2. The authors revised Fig. 3A to include bead-only control. That's nice (partially addressed). I was also hoping to see an IgG control as an essential negative control used to differentiate true, specific protein-protein interactions from false-positive background noise. It verifies that the proteins pulled down are bound by your target antibody, not the antibody itself. Not mandatory now but a definite consideration for future assays.

We thank the reviewers for this suggestion. We will adapt our protocols accordingly.

We would like to sincerely thank the reviewers, who have truly improved this manuscript. We hope that it is now suitable for publication.

© 2026. Published by The Company of Biologists under the terms of the Creative Commons Attribution License (https://creativecommons.org/licenses/by/4.0/).

Third decision letter

MS ID#: jcs.264681R2

MS Title: DHHC21 is a STIM1 protein S-acyltransferase that modulates immune function in vivo

Authors: Goutham Kodakandla; Ying Fan; Michael Zhu; Savannah West; Askar Akimzhanov; Darren Boehning

Article Type: Transfer

Dear Dr Boehning,I am happy to tell you that your manuscript has been accepted for publication in Journal of Cell Science, pending standard publication integrity checks.

© 2026. Published by The Company of Biologists under the terms of the Creative Commons Attribution License (https://creativecommons.org/licenses/by/4.0/).
